# Differing Mechanisms Underlie Sexual Size-Dimorphism in Two Populations of a Sex-Changing Fish

**DOI:** 10.1371/journal.pone.0010616

**Published:** 2010-05-12

**Authors:** Mark I. McCormick, Christopher A. Ryen, Philip L. Munday, Stefan P. W. Walker

**Affiliations:** Australian Research Council Centre of Excellence for Coral Reef Studies, School of Marine and Tropical Biology, James Cook University, Townsville, Queensland, Australia; University of Plymouth, United Kingdom

## Abstract

Variability in the density of groups within a patchy environment lead to differences in interaction rates, growth dynamics and social organization. In protogynous hermaphrodites there are hypothesised trade-offs among sex-specific growth, reproductive output and mortality. When differences in density lead to changes to social organization the link between growth and the timing of sex-change is predicted to change. The present study explores this prediction by comparing the social organisation and sex-specific growth of two populations of a protogynous tropical wrasse, *Halichoeres miniatus*, which differ in density. At a low density population a strict harem structure was found, where males maintained a tight monopoly of access and spawning rights to females. In contrast, at a high density population a loosely organised system prevailed, where females could move throughout multiple male territories. Otolith microstructure revealed the species to be annual and deposit an otolith check associated with sex-change. Growth trajectories suggested that individuals that later became males in both populations underwent a growth acceleration at sex-change. Moreover, in the high density population, individuals that later became males were those individuals that had the largest otolith size at hatching and consistently deposited larger increments throughout early larval, juvenile and female life. This study demonstrates that previous growth history and growth rate changes associated with sex change can be responsible for the sexual dimorphism typically found in sex-changing species, and that the relative importance of these may be socially constrained.

## Introduction

Sex-allocation theory suggests that the timing of sex change in sequential hermaphrodites is dependent on the relationship among sex-specific growth, reproductive output and mortality [1]–[3]. When individuals are brought together by a common requirement for limited resources, dominance hierarchies lead to the monopolisation of some resources, differential growth of individuals and the social control of sex-ratios [4], [5]. In the marine environment, resource availability can be unpredictable due to environmental patchiness and variability in population density [6]. The complex life-history of most marine organisms also means that juveniles enter social environments that may be very different from their natal state. This unpredictability has led to plasticity in the way individual fitness is maximised; individuals in different populations may change sex at different sizes and ages due to the different patterns of sex specific growth, fertility and mortality among populations [3].

In fishes there are strong links among growth, the sex of an individual and the mating system it operates within. In protogynous mating systems where males monopolise matings with many females, male reproductive success is strongly linked to size [7]. Males tend to be larger than similar aged females within the social group. This size difference can either be due to a history of faster growth in sex changing individuals [8], [9], or a product of a growth spurt that occurs coincident with sexual transition [10], [11]. While it is commonplace for males to be larger than females in a protogynous mating system [12], [13], the developmental aspects of sexual size dimorphism (SSD) have seldom been explored (see for exceptions [8], [9], [10], [14]. Indeed, this is the case not only for fishes, but for vertebrates in general [15].

Recently, the microstructural increments within otoliths (earstones) have been used to clarify the link between sex-change and growth history. Once the deposition of increments has been appropriately validated [16], the width of increments can be used as a proxy for somatic growth. Abrupt changes in increment structure, or checks, associated with key life history transitions, such as settlement [17] and sex-change [9], [10], allow a growth history to be interpreted with respect to key life events. This powerful tool gives researchers the opportunity to explore the link between growth history, sex-change and their mating system in a detail not previously possible.

The mating system adopted can depend on the density of individuals that are potential members of one or other sex. Monopolisation of resources by a small number of males may be difficult at high densities since interactions may be too frequent to allow a stable social group to form [18]–[21]. In contrast, at low densities, males may be able to visit females sufficiently often to reinforce a social hierarchy, suppress growth of females, and monopolise environmental resources and females [22]–[25]. Hence, it has been suggested that social system should strongly influence the temporal and ontogenetic relationships between sex-specific growth, sex change and SSD, and specifically, the way in which males achieve relatively larger body size [9]. We predict that growth of subordinate females should be reduced with the strength of social control by males, such that individuals rely more on accelerated growth during sex change to achieve SSD. Furthermore, at low densities the previous growth history of an individual should be less important in determining which females change sex, and its timing, because transition will be triggered by the relaxation of social control through the loss of a dominant male (e.g. [22], [26]–[28]).

The present study compares the social organisation and sex-specific growth of two populations of a protogynous tropical wrasse, *Halichoeres miniatus*, which differ in density. The social organisation of the populations is first described by examining the space use and interaction regime of individuals within the groups. Detailed examination of growth allowed the mechanisms underlying the sexual size dimorphism found in the two populations to be characterised. The presence of otolith checks associated with sex-change in this species [29] enabled an investigation of sex-specific growth in a detail not previously possible.

## Materials and Methods

### Study species and habitats

The small coral-reef wrasse *H. miniatus* is a common component of the Indo-Pacific fish fauna that inhabits the macroalgal zone and shallow reef flats. Males of this short-lived protogynous hermaphrodite are larger than females and display brightly coloured markings. Otolith increment formation has been validated as daily, and females have been experimentally shown to alter otolith accretion during sex change to form a check, which is characterised by a change in optical density and increment width [29]. Similar sex-change associated checks have been observed in the sandperch, *Parapercis cylindrica* and *P. snyderi* [9]–[11].

Study locations were located on the mid-shelf reef at Lizard Island (14°40′S 145°28′E) and the inner-shelf reef at Orpheus Island (18°37′S 146°29′E), both on the Great Barrier Reef (GBR), Australia. At Lizard Island the study population inhabited isolated patches of rubble and algae in shallow water separated by open sand flats. *Halichoeres miniatus* was common but not densely populated on the rubble patches, with an average density of 0.12 individuals/m^2^, as determined by replicate visual strip transects (20×4 m). In contrast, at Orpheus Island, the study location was part of a continuous macroalgal zone on shallow reef flat and *H. miniatus* was highly abundant in the area, with an average density of 0.45 individuals/m^2^. Both locations were situated at the leeward side of the fringing reefs, where *H. miniatus* was common. Data presented here show that at the Lizard Island location males defended non-overlapping territories containing females, whilst at Orpheus Island males were resident in specific areas and had areas of regular use, but these were seldom defended from neighbouring or transitory males. The use of the term ‘territory’ has therefore been reserved for the Lizard Island males, whilst the term ‘areas of regular use’ is used for the Orpheus Island males.

### Ethics Statement

All observations, collections and experimental procedures were approved by the James Cook University Animal Ethics Board (Approval: A1005).

### Demography and social organisation

The size and age distributions of *H. miniatus* were compared between sampled locations. Fish were collected from five sites located haphazardly around the leeward side of each island (49 individuals from Lizard Island and 69 from Orpheus Island). Fish were collected using a monofilament fence net, a clove oil/seawater solution (in a spray bottle) and hand-net, held in 15 l plastic bags for up to 30 min and killed by an overdose of clove oil/seawater solution once back aboard the research boat. Age was determined by counting the increments in the transverse sections through the nucleus of one sagittal otolith from each fish, prepared using the protocol of Wilson and McCormick [30]. Sex for each individual was initially determined by the colour patterns (terminal or initial phase) and then by macroscopic examination of their gonads under a dissection microscope. Testes were identified by their smooth surface and cream colouration while ovaries were identified by their yellow colouration and a rough surface texture, indicating the presence of developed eggs [31].

To determine the social organisation of *H. miniatus* at each study location, behavioural observations were made on all males and the largest females in a group of fish from each location (7 females and 7 males at Lizard Island, 6 females and 7 males at Orpheus Island). To facilitate recording the location and movement of individuals, areas were mapped with the aid of a reference grid of nylon string at both study locations (2.5 m square grids: 15×25 m at Lizard Island; 15×30 m at Orpheus Island). Males and females within the grids were collected using hand nets and a dilute clove oil/seawater solution, transferred to a small clip-seal bag containing seawater and measured with callipers (standard length (SL), mm). To facilitate individual identification all the males and the largest females were tattooed subcutaneously near the dorsal fin with a fluorescent elastomer (Northwest Marine Technologies) using a 27 gauge hypodermic needle while restrained by the plastic bag. During the tagging process fish were partially sedated due to the anaesthetic clove oil used in capture. This method of tagging minimised stress and scale damage through handling and could be done underwater to minimise processing time [32]. Tagging left a 0.5 to 0.8 mm long mark (on a 45 to 90 mm SL fish) and has been shown not to influence growth or mortality of reef fishes [32]. Recovery was rapid (∼30 sec) and fish were released at the point of capture. Upon release fish quickly returned to their areas of residence and males resumed territorial behaviour.

Behavioural observations began the day after tagging and were made over three days for five hours per day. Male/female interactions (displays, chases, physical contact), feeding (strikes to the substrate), movement and spawning were recorded. Each observation period followed one individual for 15 minutes and all interactions were recorded during that time. A scuba diver followed individuals at a distance of 2–3 m and the proximity of the diver did not appear to influence fish behaviour. Behaviour was compared between Orpheus and Lizard Island populations with a one-factor MANOVA, with behavioural categories used as the response variables. The nature of significant differences in behaviour found by MANOVA was explored using t-tests. At the end of the study, tagged fish were recollected and euthanised using the previously mentioned protocol to allow age determination from increments in otolith cross sections.

The location and movement of tagged individuals was plotted on a scale map of the study areas. Home range sizes and the degree of overlap of home ranges for males and the largest females were measured and compared between the two locations using a one-way analysis of variance (ANOVA). Residual analysis was used to test whether data conformed to the assumptions of homogeneity of variance and normality.

### Ontogeny, growth and SSD

Microstructural increments on the sagittal otoliths were used to describe the growth history of the tagged fish. At the end of the observation period, fish were recaptured (as above), and euthanised by cold shock in a slurry of seawater and crushed ice to minimise stress. Sagittal otoliths were processed to produce a transverse section of the distal-rostral plane, following the methods of Wilson and McCormick [30]. Digital images were taken at a 400× magnification and an image analysis program (Optimus 6.5) was used to measure the distances between the daily otolith increments along the primary growth axis. Multiple regression was used to confirm an age-independent, predictive relationship between otolith growth and somatic growth (i.e., age was used as a covariate). The body size- and otolith size- (maximum otolith radius, MOR) distributions of females and males were compared within each population using t-tests, and sex-specific otolith increment width profiles were used to infer the timing and shape of growth divergence. No attempt was made to compare the magnitude of otolith growth between populations since different relationships exist between somatic growth and otolith growth between populations.

Increment width profiles were compared between sexes (male, female), locations (Orpheus and Lizard Islands) and between initial larval growth and juvenile growth using a three-factor repeated measures MANOVA. Two ten-day periods were chosen to typify early larval and juvenile growth (day 1–10 and day 100–109 respectively). Pillai's trace was used as the test statistic for within subject (i.e., consecutive increment widths) effects and their interactions [33]. Significant terms were interpreted from increment graphs.

To explore whether there was an increase in increment width (as a proxy for somatic growth) associated with sex change, the otoliths of males were re-examined and increment widths were re-plotted so that they were centred on the check in the otolith associated with sex-change [29]. This makes it easier to distinguish changes in otolith growth that occur at the time of sex-change and avoids the problem of masking through the averaging of increment widths of fish that undergo the transition at a variable age. Increment widths were compared from 20 days before and after the check using repeated measures MANOVA.

We explored variation in both the age at sex change and the size at sex change between populations using Kolmogorov-Smirnov two-sample (K–S) tests. The age at sex change for each individual was determined by counting the number of increments from the otolith nucleus to the sex change-associated check mark. The size at sex change for each individual was back-calculated using the biological intercept method [34]. The size at hatching was estimated as the mean larval size at hatching of a congeneric, *Halichoeres poecilopterus* [35]. For both populations a linear model was found to best describe the otolith radius versus body size relationship.

## Results

### Population demography

**Figure 1:**
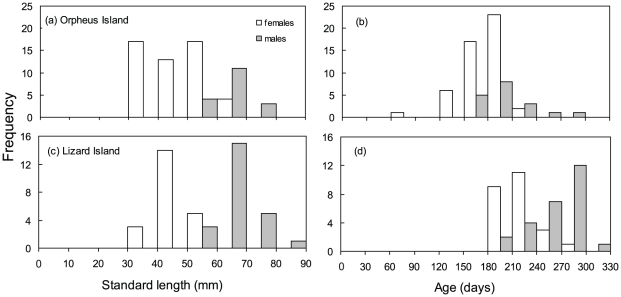
Comparison of sex-related size and age distributions. Size (a, c) and age (b, d) distributions for two populations of *Halichoeres miniatus* at Orpheus Island (a, b) and Lizard Island (c, d). Females are shown as white bars and males as grey.

A total of 69 fish were collected from Orpheus Island (51 female and 18 male), and 49 fish from Lizard Island (23 female and 26 male). The size and age and size-at-age distributions for both locations were characteristic of a protogynous species (Fig. 1), with no males in the smaller size and younger age classes. Examination of the gonads also revealed no initial phase males, suggesting that *H. miniatus* in these populations are monandric (i.e. males exclusively derived from females) protogynous hermaphrodites. Overall, there was more overlap in the frequency distributions of female and male age than size. Males and females at Orpheus Island showed a greater overlap in size and age classes than at Lizard Island indicating greater variability in the size and age at sex change in the Orpheus Island population. The average male size at Orpheus Island was 65.6 mm SL and 71.1 mm SL at Lizard Island, the smallest males being 55.7 mm SL and 60.0 mm SL respectively. Average female sizes were 48.4 mm SL and 49.5 mm SL at Orpheus and Lizard Island, respectively. Several females were larger than the smallest males at Orpheus Island but males were always larger than females at Lizard Island. The average age of males at Orpheus Island was 200 days (youngest = 152 days) and at Lizard Island was 263 days (youngest = 196 days), while the average age of females was 178 days and 193 days at Orpheus and Lizard Island, respectively. At both locations no individuals were over 365 days indicating that, at these locations, *H. miniatus* is an annual species.

### Social structures

**Figure 2:**
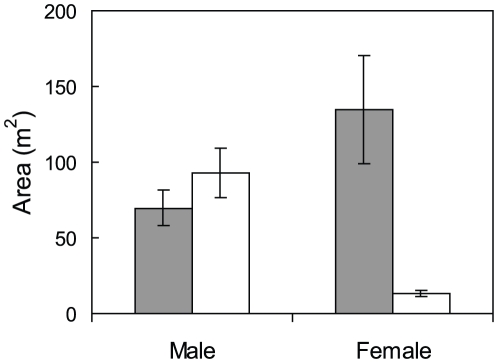
Comparison of areas of regular use between sexes and locations. Mean areas of regular use for male and female *Halichoeres miniatus* (± SE) for Orpheus Island (grey bars) and Lizard Island (white bars) sampling locations (n = 7, except 6 females at Orpheus Island).

Females at Orpheus Island used larger areas on average than females at the Lizard Island location (134.4 m^2^ and 13.2 m^2^ respectively; t = −4.38, df = 27, *P*<0.0001; Fig. 2). In contrast, males had similar mean areas of use at both locations (69.8 m^2^ at Orpheus Island and 92.8 m^2^ at Lizard Island; t = 1.10, df = 18, *P* = 0.286; Fig. 2). Females at the Orpheus Island location were far less site attached than at the Lizard Island location and moved through multiple male areas of use on a regular basis. At Lizard Island, the females remained within their territories and were relatively isolated from similar-sized females. There was a large amount of overlap (average of 43.6%) between male areas of regular use at the Orpheus Island location, and perimeters were not rigorously maintained. In contrast, males at the Lizard Island location had territories with no overlap (i.e. 0%) and perimeters that were defended aggressively from other males, as was access to females within the territory.

**Figure 3:**
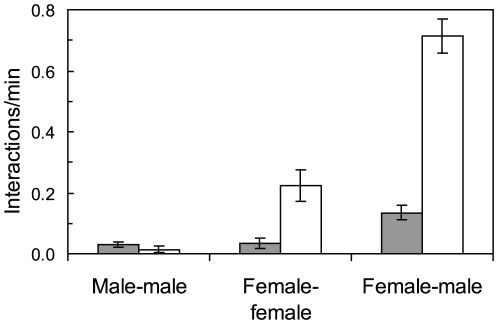
Comparison of males and female encounter rates between locations. Frequency of encounters per minute (± SE) for *Halichoeres miniatus* at Orpheus Island (grey) and Lizard Island (white) sampling locations. Based on the 15 min observations of 7 males and 7 females at each location.

The behaviour of *H. miniatus* differed between the two locations (MANOVA: Pillai's trace = 0.699, df = 2, *P*<0.0001). Male and female interaction rates differed between locations (Fig. 3). Males interacted with females 26-times more often at the Lizard Island location (0.713 interactions/min) compared to the Orpheus Island location (0.027 interactions/min). The largest females interacted with other females six times more often at the Lizard Island location than at the Orpheus Island location (0.219 interactions/min and 0.035 interactions/min respectively). There was higher male to male encounter rate at Orpheus Island than at Lizard Island (t = 4.04, df = 45, *P*<0.0001; Fig. 3), and there were distinct differences in the male behaviour between locations. Male encounters were always highly aggressive at Lizard Island while at Orpheus Island encounters were characterised by displays and few chases or contact. There was no significant difference in the feeding rates of males and females between the two locations (two-way ANOVA: F_1,90_ = 3.28, *P* = 0.073), with an average feeding rate of 1.81 bites/min for females and 0.979 bites/min for males.

### SSD, growth, and sex change

Consistent with the broader demographic patterns described above, males from the behavioural focal sample were larger than females on average at both Orpheus Island (female = 48.2 mm SL, male = 65.6 mm SL, t = 7.9, df = 66, *P*<0.01) and Lizard Island (female = 47.5 mm SL, male = 68.4 mm TL, t = 11.09, df = 47, *P*<0.01). Differences in the sex-specific body size distributions were reflected in the sex-specific otolith size distributions at both Orpheus Island (mean otolith radius: female = 855 µm, male = 1064 7 m, t = 6.5, df = 66, *P* = 0.01) and Lizard Island (mean otolith radius: female = 782 µm, male = 936 µm, t = 5.37, df = 47, *P*<0.01). Including otolith size into a linear regression model between age and body size increased the resolution of the model in predicting body size among both the Orpheus Island individuals (multiple regression: F_(2,65)_ = 116.72, *P*<0.01, r^2^ = 0.78; partial correlation coefficient, Age = 0.1, Otolith size = 0.8) and Lizard Island individuals (multiple regression: F_(2,46)_ = 27.58, *P*<0.01, r^2^ = 0.56; partial correlation coefficients, Age = 0.2, Otolith size = 0.37). Otolith size was a positive predictor of body size, independent of age, at both locations.

**Figure 4:**
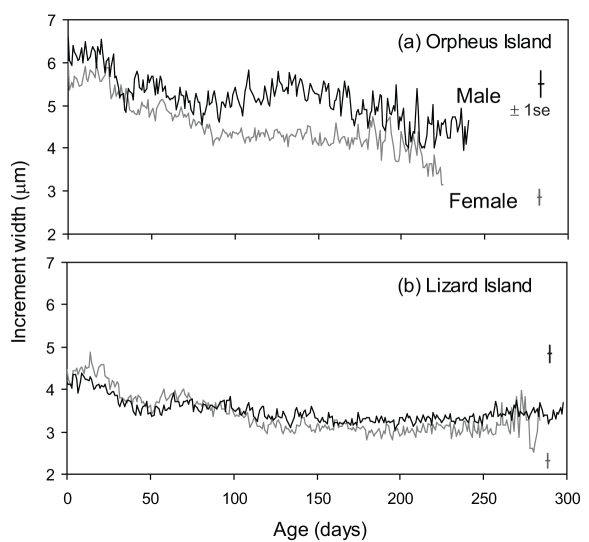
Growth of males and females by location. Comparison of mean daily increment widths of male (i.e. females that changed sex to males; black) and female (i.e. non-sex changing fish; grey) *Halichoeres miniatus* collected from (a) Orpheus Island (n = 18 males, 48 females) (b) and Lizard Island (n = 16 males, 23 females). Mean standard errors are inset.

Comparisons of the growth estimates derived from the otolith increments revealed differences in the average daily increments between sexes that differed between locations (Location x Sex interaction: F_1,1989_ = 12.13, *P* = 0.0006; Fig. 4). At the Orpheus Island location those females that had changed sex into males generally had wider increment widths than females that did not change sex (Fig. 4a), but this was not the case in the Lizard Island population (Fig. 4b). There was an overall significant effect of life stage on the trend (mean±se; 1–10 d, 5.1±0.06; 100–109 d, 4.1±0.06; F_1,1989_ = 108.51, *P*<0.0001), but no significant interactions between life stage and sex or location (*P*>0.05). Overall, these findings suggest that early growth history is influencing which individuals will change sex later in life in the Orpheus Island population, but not in the Lizard Island population.

**Figure 5:**
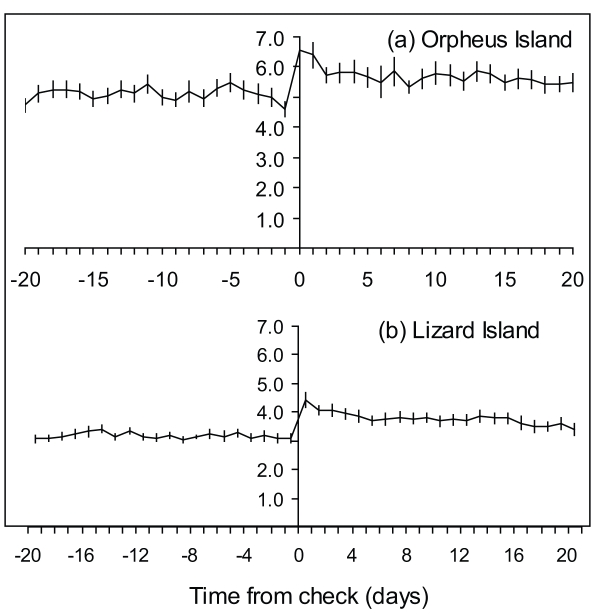
Changes in male growth at sex change. Comparison of mean (± SE) otolith increment width (microns) profiles of males *Halichoeres miniatus* centred on the check-mark associated with sexual transition for fish collected from (a) Orpheus Island (n = 18) and (b) and Lizard Island (n = 16).

**Figure 6:**
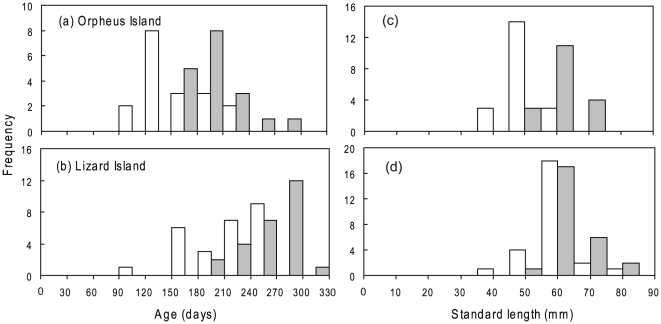
Size and age at sex change. Frequency distributions of age (a, b) and standard length (c, d) at sex-change to males (determined from check marks) compared to the distributions of male *Halichoeres miniatus* at collection from (a, c) Orpheus Island and (b, d) Lizard Island. Age and size distributions of males at collection shown in grey, while age and back-calculated size distributions at sex change (from the otolith check marks) are displayed in white.

Centring the increment width profiles on the check associated with sex-change highlights that the increase in increment widths later in life is associated with sex-change (Fig. 5). In males from the Lizard Island location (Fig. 5b), increment widths rapidly increase in association with sex-change (repeated measures MANOVA: F_(26, 13)_ = 21.867, *P*<0.0001). Although this increased otolith growth is still evident in the Orpheus Island population (repeated measures MANOVA: F_(18, 21)_ = 5.8937, *P* = 0.0001, Fig. 5a), the growth is more variable for the Orpheus Island location. The age of check mark occurrence also differed between the two locations (K–S test, *P*<0.001; Fig. 6a, c). The average age of check occurrence at Orpheus Island was 158 days and at Lizard Island was 209 days. The age distributions of the check marks were also skewed in the opposite direction: the Lizard Island population exhibited a negative skew, while the Orpheus Island population displayed a positive skew. The back-calculated size at sex change also differed between locations (K–S test, *P*<0.001; Fig. 6 b, d). Females in the Orpheus Island population on average changed sex to males at a smaller size than females in the Lizard Island population.

## Discussion

Like other polygynous animals, polygynous reef fishes are typically sexually size-dimorphic, which can be explained by the high level of sexual selection acting on male body size [13]. However, their complex reproductive life histories, which frequently involve sex-change from female to male, raises an important question: how is SSD achieved, and how do individuals overcome the conflict between sex-specific body size trade-offs (implicit in the size-advantage model for protogynous sex change [7]) and a sequentially hermaphroditic life history? Here, we have illustrated that the mechanism for SSD is labile, and suggested that the growth mechanism that underlies SSD may be related to the social system and the level of behavioural dominance that operates within that system.

Social organisation and mating system are influenced by the physical (e.g. resource) and behavioural (e.g. competitor) environment that individuals within a group experience [21], [36]. In turn, the composition of the assemblage within which an individual interacts can affect the potential for resource and female defence, and thus the ways in which individuals and sexes can optimise their life history within phylogenetically determined limits [20], [37], [38]–[43]. The present study describes the occurrence of two very different social organisations in the same species and their consequences for growth, sex-change, and SSD. At the densely populated Orpheus Island location, female *H. miniatus* roamed throughout multiple male territories and encountered multiple males and females on a regular basis. Males maintained loosely defined defended areas, which could overlap with the space used by other males, and encounters were not overly aggressive. This social system appears to be a loose form of resource (probably shelter or food) defence polygyny, with the potential for female choice of males. In contrast, the females at the low density Lizard Island location maintained small territories within large male territories and defended them from other females. Females did not cross between male territories and only encountered a single male on a regular basis. Males at Lizard Island actively defended large territories encompassing multiple females from other males. There was no overlap between male territories and male encounters were always highly aggressive. These observations suggest a strict, hierarchically organised haremic society (i.e. female defence polygyny) with strong internal control of space use.

Density can affect the relationship between growth history and the occurrence and timing of sex-change through its influence on social organisation. A recent study comparing four species of wrasse that exhibit different social systems illustrates that the relationship between growth and sex change can vary with the strength of the dominance interactions [44]; the stronger the dominance network, the weaker the influence of early growth history on SSD and prior growth trajectories on which individuals change sex within the population. The present study suggests that the same pattern can occur within an individual species when groups are exposed to different environmental and behavioural regimes. The population at Orpheus Island had a loose social structure and the females that subsequently changed sex were those that acquired and maintained an initial size advantage early in life. Previous growth history, in addition to sex-change associated growth acceleration, drove the pattern of SSD at Orpheus Island. In contrast, females and males from the Lizard Island population showed little difference in growth rate until later in life; initial growth advantages did not carryover and growth acceleration following sex change induction was the sole mechanism by which SSD was achieved.

The present study illustrates the mechanisms by which SSD is achieved in sequential protogynous hermaphrodites. In both the high and low density populations, growth acceleration coincident with sexual transition was integral to achieving SSD. These findings are similar to two recent studies of tropical protogynous sandperch (*Parapercis* sp; Pinguipedidae) [9], [10]. Irrespective of previous growth and social system, sex change associated growth acceleration would benefit the sex-changer due to the advantage of large body size in female- and resource-defence competition. However, the advantage of rapid growth prior to sex change (as a strategy for becoming a large dominant male) is likely to be dependent on social structure. Rapid growth during the juvenile and female phase may be selected against within a strict dominance hierarchy due to social constraints imposed by dominant individuals. Dominants may limit rapid growth in subordinates through the direct control over food resources, or through the threat of punishment and group eviction [5], [45], [46]. When individuals recruit to a hierarchically organised social group, it may be more advantageous for those individuals to limit growth, remaining smaller than their immediate dominant, hence avoiding conflict over rank in a resource-limited environment [4], [5].

Average otolith growth was found to differ markedly between the two locations separated by four degrees of latitude. This was expected given the many differences between locations that may contribute to growth differences (e.g. [47], [48]). These include: differences in the environment, such as temperature or light regime and food availability (quality and quantity) [49], [50]; differences in the levels of physiological stress associated with environmental or biological stress (e.g. habitat quality, composition of the interacting community, rates of parasitism [51], [52]); differences in the selection pressure for particular growth phenotypes driven by differences in the predator community [53], [54]; differences in physiological optima for spatially separated populations [55]; or simply differences in the otolith versus somatic growth relationships [56]. While the impact of these potential differences between the sampled locations is unknown, they do not influence the differences found within populations in the mechanisms that lead to SSD, which was the goal of the study.

In the present study we have demonstrated that sexual dimorphism can arise through differential growth by two mechanisms: a growth spurt coincident with sex-change; accumulation of a historical growth advantage for sex-changing individuals; or a combination of both. We suggest that the social organisation of a group will determine the relative importance of previous growth history, and that advantages of previous high growth may only be realised when social control is relaxed; as shown at high population densities. Importantly, the link between social structure, age-based growth and SSD illustrates the flexible nature of growth and its relationship to sex change in protogynous fishes. The initial growth advantages evidenced here may be due to genetic or non-genetic parental (particularly maternal) effects, which have recently been shown to carryover into the juvenile phase to influence growth and survival [57]–[59]. Our findings suggest that we should be targeting the level of individual groups if we are to obtain a detailed understanding of the link between physical and behavioural environments, growth history and sex-change.
